# The Perivascular Fat Attenuation Index Improves the Diagnostic Performance for Functional Coronary Stenosis

**DOI:** 10.3390/jcdd9050128

**Published:** 2022-04-23

**Authors:** Hankun Yan, Na Zhao, Wenlei Geng, Zhihui Hou, Yang Gao, Bin Lu

**Affiliations:** Department of Radiology, Fu Wai Hospital, National Center for Cardiovascular Diseases, Chinese Academy of Medical Sciences, Peking Union Medical College, Beijing 100037, China or yanhankun@fuwaihospital.org (H.Y.); 15810686235@163.com (N.Z.); sd785291190@gmail.com (W.G.); konglingyihui@126.com (Z.H.)

**Keywords:** coronary artery disease, coronary computed tomography angiography, ischemia, fat attenuation index

## Abstract

**Background:** Coronary computed tomography angiography (CCTA) is an established first-line test in the investigation of patients with suspected coronary artery disease (CAD), while the perivascular fat attenuation index (FAI) derived from CT seems to be a feasible and efficient tool for the identification of ischemia. The association between the FAI and lesion-specific ischemia as assessed by fractional flow reserve (FFR) remains unclear. **Methods:** In a total of 261 patients, 294 vessels were assessed for CCTA stenosis, vessel-specific FAI, lesion-specific FAI, and plaque characteristics. The diagnostic accuracies of each parameter and the combined approach were analyzed via the receiver operating characteristic curve (ROC) with FFR as the reference standard. The determinants of FAI were statistically analyzed. **Results:** The cutoff values of vessel-specific FAI and lesion-specific FAI scores calculated according to the Youden index were −70.97 and −73.95 HU, respectively. No significant differences were noted between them; however, they exhibited a strong correlation. No significant differences were noted between the area under the curve (AUC) scores of vessel-specific FAI (0.677), lesion-specific FAI (0.665), and CCTA (0.607) (*p* > 0.05 for all) results. The addition of two FAI measures to the CCTA showed improvements in the discrimination (AUC) and reclassification ability (relative integrated discrimination improvement (IDI) and category-free net reclassification index (NRI)), vessel-specific FAI (AUC, 0.696; NRI, 49.6%; IDI, 5.9%), and lesion-specific FAI scores (AUC, 0.676; NRI, 43.3%; IDI, 5.4%); (*p* < 0.01 for all). Multivariate analysis revealed that low-attenuation plaque (LAP) volume was an independent predictor of two FAI measures. **Conclusion:** The combined approach of adding vessel-specific FAI or lesion-specific FAI scores could improve the identification of ischemia compared with CCTA alone. The LAP volume was the independent risk factor for both tools.

## 1. Introduction

Coronary artery disease (CAD) remains the leading cause of death in upper-middle and high-income economies [1]. Coronary computed tomography angiography (CCTA) is an established first-line test in the investigation of patients with suspected CAD, because it has a high negative predictive value and high accuracy in the diagnosis and exclusion of CAD [2,3,4]. However, only about half of obstructive coronary stenosis cases lead to ischemia [5,6] and are associated with worsened survival [7]. Therefore, it is important to find other factors beyond coronary stenosis to improve the recognition of lesion-specific ischemia.

The perivascular adipose tissue (PVAT) interacts with the arterial wall in a bidirectional manner and has been associated with the process of atherogenesis characterized by calcification and adverse clinical prognosis [8], which implies that there might be a common inflammatory background between PVAT and calcification. The perivascular fat attenuation index (FAI) is a metric derived from CT that captures the balance between lipids and water within PVAT. The FAI is associated with an increased risk of all-cause and cardiac mortality and adverse clinical outcomes [9], and could also help in detecting the presence of culprit lesions in patients with acute myocardial infarction [10,11]. Higher lesion-specific FAI and vessel-specific FAI values are linked to hemodynamically significant stenosis [12,13,14,15]. However, studies on the relationship between lesion-specific FAI and vessel-specific FAI are still inadequate. This study hypothesizes that there is a high degree of consistency between lesion-specific FAI and vessel-specific FAI, and that both of them can improve the diagnostic performance of CCTA in identifying lesion-specific ischemia. Accordingly, this study aims to investigate the relationship between lesion-specific FAI and vessel-specific FAI, as well as the associations between them and with coronary stenosis severity and ischemia using fractional flow reserve (FFR) as a reference standard.

## 2. Materials and Methods

### 2.1. Study Population

In this study, we retrospectively collected data from patients with suspected or known stable CAD from January 2012 to March 2020 at our institution. The inclusion criteria were as follows: (1) patients with angina or angina-equivalent symptoms; (2) the presence of at least one lesion with stenosis diameters ranging between 30% and 90% of the major epicardial vessels (diameter ≥ 2 mm) based on CCTA; (3) patients who underwent CCTA, invasive coronary angiography (ICA), and FFR measurements within 2 weeks. The exclusion criteria were as follows: (1) age < 18 years; (2) previous history of myocardial infarction; (3) previous history of coronary revascularization; (4) insufficient quality of CCTA images; (5) patients with anatomic variations in the heart or coronary arteries. This study was approved by the Institutional Review Board of our institution, and informed consent from all patients in this study was waived.

### 2.2. Coronary Computed Tomography Angiography Acquisition

All CCTA scans were performed using a dual-source CT scanner (Definition Flash, Siemens Healthineers, Forchheim, Germany). All patients were scanned by prospective electrocardiogram (ECG) gating technology, and images were acquired at 35–75% of the R–R interval. Beta-blockers were administered when the heart rate > 75 beats/min. Sublingual glyceryl trinitrate was administered before scanning in all patients. The scanning parameters were shown as follows: tube voltage, 100 kV or 120 kV (according to the body mass index of patients); tube current, automatic tube current modulation; rotation time, 0.28 s per rotation; slice thickness, 0.75 mm; gap, 0.70 mm. The optimal cardiac phase was selected by radiology technicians. During scanning, 60–70 mL of contrast medium (Iohexol, Shuangbei 350; Beilu Pharmaceutical Co., Ltd., Beijing, China) was injected into the antecubital vein through a dual-cylinder high-pressure syringe (Stellant; Medrad, Indianola, PA, USA) at a speed of 4.5–5.0 mL/s and then flushed with 30–40 mL of saline at the same speed. The calcification score (CS) of the coronary artery was measured as previously described by Agatston [16]. Stenosis severity was categorized as 30–49%, 50–69%, and 70–90% in coronary segments ≥ 2 mm by two experienced local radiologists who were blinded to the patient’s condition. When there were different opinions, a consensus was drawn after discussion. Coronary stenosis ≥ 50% was considered as obstructive stenosis.

### 2.3. Coronary Plaque Analysis

Plaque areas > 1 mm^2^ in coronary segments ≥ 2 mm were measured with a semi-automated dedicated plaque analysis software (Coronary Plaque Analysis, version 2.0, Siemens Healthineers, Forchheim, Germany). The quantitative plaque components were automatically generated according to scan-specific thresholds within the manually designated area. Adjustments were performed if necessary. The remodeling index, plaque length, total plaque volume (TPV), low-attenuation plaque (LAP), intermediate-attenuation plaque (IAP), and calcified plaque (CP) values were previously measured. The remodeling index is the ratio of the largest vessel diameter at the lesion site to the vessel diameter at the proximal reference point, while a remodeling index > 1.1 indicates positive remodeling (PR) [17]. Two experienced observers performed the analyses and the average values were used for further analysis.

### 2.4. Perivascular Fat Attenuation Index Acquisition

Coronary PVAT around the epicardial coronary arteries with an attenuation window of −190 to −30 Hounsfield units (HU), is defined as the adipose tissue located within a distance from the outer vessel wall equal to the diameter of the adjacent coronary vessel [10]. Perivascular FAI was defined as the mean CT attenuation of PVAT, and it was also performed by a semi-automated post-processing software (Coronary Plaque Analysis, version 2.0, Siemens Healthineers, Forchheim, Germany). Vessel-specific FAI values were acquired as described previously. To avoid the effects of the aortic wall, the most proximal 10 mm of the right coronary artery (RCA) was excluded and the proximal 10–50 mm of the vessel was analyzed [9]. In the left anterior descending artery (LAD) and left circumflex artery (LCX), the proximal 40 mm of each vessel was analyzed. Lesion-specific FAI scores for lesion plaques interrogated with FFR evaluation were measured from the proximal to the distal shoulder of the lesion. Additional manual optimization was performed, if necessary, to avoid the effects of non-adipose tissues, such as the small side branches or coronary veins. The time periods required for vessel-specific FAI and lesion-specific FAI analyses are between 6 and 8 min and between 3 and 5 min, respectively. Vessel-specific FAI and lesion-specific FAI scores of 30 consecutive vessels were measured by two experienced radiologists who were blinded to clinical, CCTA, and FFR data to evaluate the reproducibility between observers.

### 2.5. Invasive Coronary Angiography and Fractional Flow Reserve Measurements

Invasive FFR measurements were assessed during the ICA inspection, and all operations were performed by a cardiovascular physician with extensive work experience. ICA and FFR were performed according to standard practices, as previously described [18]. FFR was the ratio of the pressure of the distal coronary artery to the aortic pressure during the maximum hyperemia, and it was measured by using a 0.014 inch pressure guidewire (St Jude Medical Systems, Minneapolis, MN, USA). The FFR was measured at approximately 2 cm distal to the lesion stenosis. A hyperemic state was induced by continuous administration of intravenous adenosine at a rate of 160 mg/kg/min. Invasive FFR ≤ 0.80 was considered that the stenosis was physiologically significant and causal of lesion-specific ischemia [19,20].

### 2.6. Statistical Analysis

Continuous variables were presented as means ± standard deviation (SD) in the case of normal distribution and medians (interquartile range) in the case of non-normal distribution, while categorical variables were expressed as numbers and percentages. To evaluate interobserver reproducibility, intraclass correlation coefficients were used to evaluate the interobserver variability of vessel-specific FAI and lesion-specific FAI scores. The receiver operator characteristic curve (ROC) was created to predict the area under the curve (AUC), while *p*-value, diagnostic accuracy, sensitivity, specificity, positive predictive value (PPV), and negative predictive value (NPV) scores were calculated with a 95% confidence interval (CI), using invasive FFR as the reference standard. The AUCs of different methods were compared using the method previously described by Delong et al. [21]. The accuracy, sensitivity, and specificity of different methods were compared using Cochran’s Q test, then the post-Dunn test and Bonferroni correction were used for intergroup comparison [22], and PPV and NPV were compared using a chi-square test. Additive values of vessel-specific FAI and lesion-specific FAI measures were evaluated by relative integrated discrimination improvement (IDI) and category-free net reclassification index (NRI) scores [23,24]. The best cutoff values of vessel-specific FAI and lesion-specific FAI scores were selected according to the Youden index (defined as %sensitivity + %specificity − 1). Data were compared using Student’s *t*-test, Mann–Whitney U test, Kruskal–Wallis test, or chi-square test as appropriate. Univariable and multivariable logistic regression analyses were performed to determine predictive factors of vessel-specific FAI and lesion-specific FAI, respectively. All statistical analyses were performed with SPSS 25.0 (IBM Corp., Armonk, NY, USA), MedCalc 19.0.4 (MedCalc Software, Ostend, Belgium), and R 3.3.3 (R Foundation for Statistical Computing, Vienna, Austria) software. Statistical tests were two-tailed, and *p*-values < 0.05 indicated statistical significance.

## 3. Results

### 3.1. Clinical Characteristics

**Figure 1:**
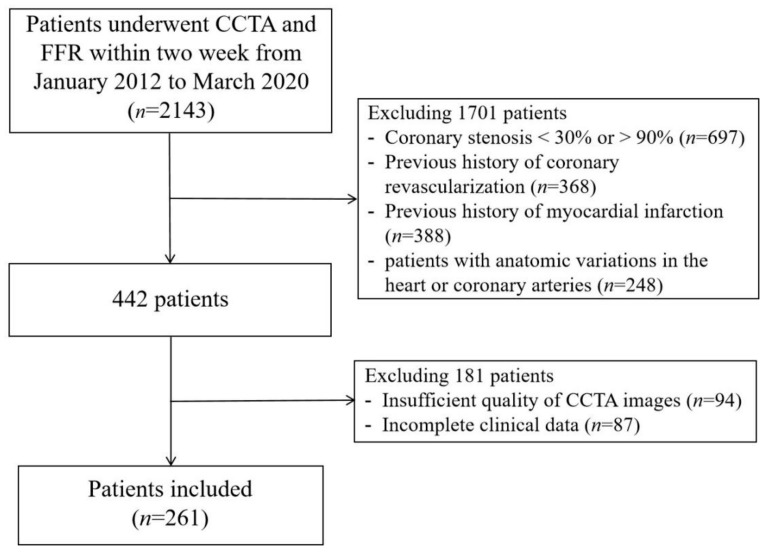
Flowchart of patient’s selection. CCTA, coronary computed tomography angiography; FFR, fractional flow reserve.

The flowchart of patient selection is shown in Figure 1. Finally, a total of 261 patients were included in this study, in whom 294 vessels were interrogated by invasive FFR, including LAD (210; 71.4%), LCX (35; 11.9%), and RCA (49; 16.7%), respectively. The baseline characteristics of the patients are shown in Table 1. The mean age of the patients was 56.3 ± 8.7 years, and men accounted for 72.0% (188) of patients in this study. Lesion-specific ischemia was found in 45.2% (133/294) of vessels in 44.1% (115/261) of patients by obtaining FFR as a reference; the mean FFR value was 0.80 ± 0.11. There were 206 (70.8%) vessels with obstructive stenosis based on CCTA results.

### 3.2. Discrimination of Ischemia

**Figure 2:**
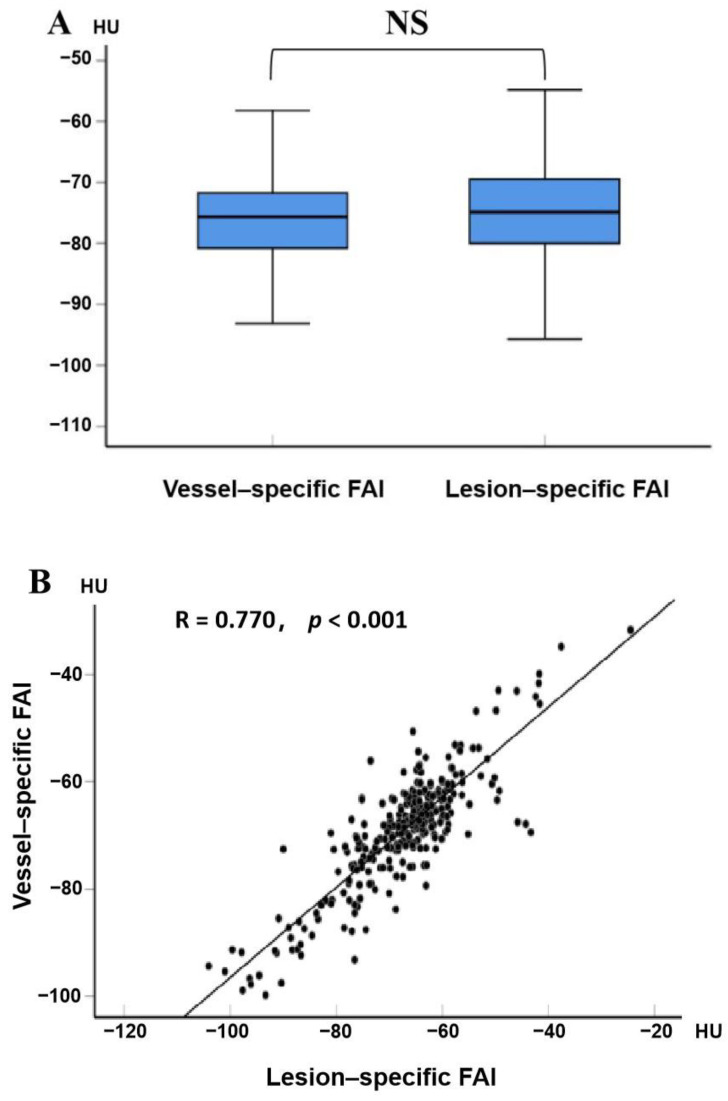
Comparisons of vessel-specific FAI and lesion-specific FAI scores: (**A**) the distribution of vessel-specific FAI and lesion-specific FAI scores, with medians, quartiles, and ranges shown in the box plot; (**B**) the correlation between vessel-specific FAI and lesion-specific FAI scores, with the scatter diagram showing a positive correlation between them (R = 0.770, *p* < 0.001). FAI, fat attenuation index; HU, Hounsfield unit; NS, non-statistical significance.

The intraclass correlation coefficients were shown as follows: vessel-specific FAI, 0.98 (95% CI, 0.95–0.99); lesion-specific FAI, 0.91 (95% CI, 0.62–0.97). The distribution of vessel-specific FAI and lesion-specific FAI scores is shown in Figure 2. There were no significant differences between vessel-specific FAI and lesion-specific FAI scores (*p* = 0.135), and they demonstrated a strong, almost collinear association (R = 0.770, *p* < 0.001).

**Figure 3:**
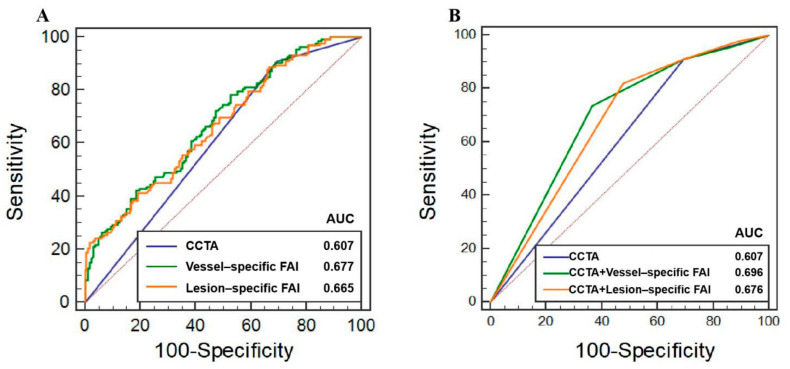
Receiver operating characteristic curves for the CCTA, vessel-specific FAI, and lesion-specific FAI in predicting ischemia: (**A**) ROC curves for predicting ischemia using CCTA, vessel-specific FAI, and lesion-specific FAI. (**B**) ROC curves of models using CCTA with and without vessel-specific FAI and lesion-specific FAI, respectively. Cutoff values of −70.97 HU for vessel-specific FAI and −73.95 HU for lesion-specific FAI according to the Youden index were used for the comparison between CCTA with and without these scores. CCTA, coronary computed tomography angiography; FAI, fat attenuation index; ROC, receiver operating characteristic curves; HU, Hounsfield unit.

**Figure 4:**
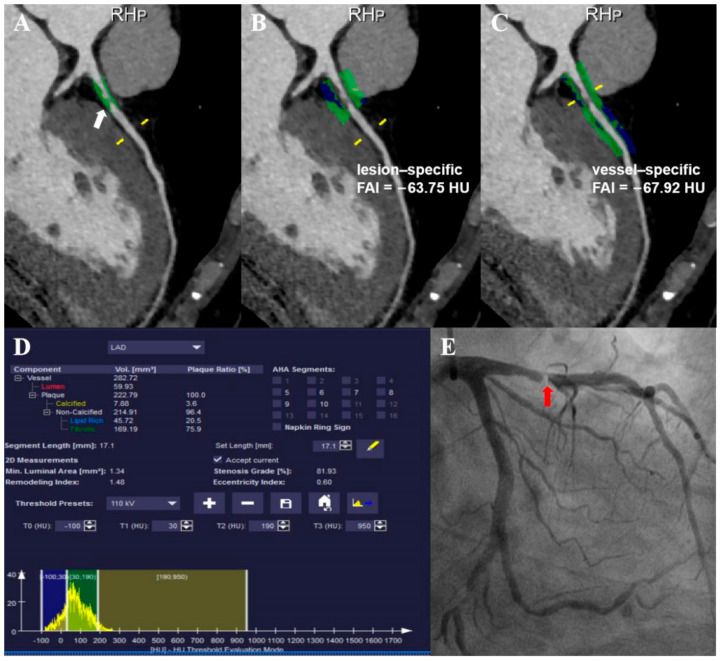
Example of a 42-year-old man with chest pain: (**A**) CCTA image showed a lesion analyzed with dedicated plaque analysis software proximal to the LAD with stenosis ranging between 70 and 90% (white arrow); (**B**,**C**) color-coded CPR image reveals that the mean perivascular FAIs of the lesion (lesion-specific FAI) and proximal 40 mm of LAD (vessel-specific FAI) were −63.75 HU and −67.92 HU, respectively; (**D**) measurement list shows the contents of various plaque components; (**E**) ICA showed that the stenosis degree of the lesion was about 90% (red arrow), and then FFR confirmed that the stenosis was hemodynamically significant (FFR = 0.75). CCTA, coronary computed tomography angiography; FAI, fat attenuation index; LAD, left anterior descending artery; CPR, curved planar reformation; HU, Hounsfield unit; ICA, invasive coronary angiography; FFR, fractional flow reserve.

Per-vessel AUCs for CCTA, vessel-specific FAI, and lesion-specific FAI scores were 0.607 (95% CI, 0.549–0.663), 0.677 (95% CI, 0.620–0.730), and 0.665 (95% CI, 0.608–0.719), respectively (Figure 3). According to the Youden index, the optimal cutoff values for vessel-specific FAI and lesion-specific FAI scores were −70.97 HU and −73.95 HU, respectively. Table 2 provides measures of diagnostic characteristics. Although the AUC of the vessel-specific FAI was higher than other methods, there were no statistically significant differences between them; the difference in AUC for the lesion-specific FAI was 0.012 (95% CI, −0.033–0.057, *p* = 0.612), and for the CCTA was 0.070 (95% CI, −0.004–0.143, *p* = 0.062). The difference in AUC of the lesion-specific FAI was 0.058 (95% CI, −0.015–0.131, *p* = 0.118) higher than CCTA, but there was still no statistically significant difference between them. No statistically significant differences in accuracy between CCTA, vessel-specific FAI, or lesion-specific FAI (all *p* > 0.05) were noted. The sensitivity of the CCTA and lesion-specific FAI methods were both higher than that of the vessel-specific FAI (*p* = 0.001, *p* = 0.011, respectively), while there were no statistically significant differences between CCTA and lesion-specific FAI (*p* = 1.000). The specificity of the vessel-specific FAI was higher than that of CCTA and lesion-specific FAI (*p* = 0.002, *p* = 0.013, respectively), and the difference between the CCTA and lesion-specific FAI was not statistically significant (*p* = 1.000). There were no statistically significant differences in NPV and PPV among them (*p* = 0.825, *p* = 0.356, respectively). A representative case is given in Figure 4.

### 3.3. Additive Values of FAI to CCTA

Figure 3 presents the ROCs for the three models, while the AUC, category-free NRI, and relative IDI values for the three models are shown in Table 3. Compared with the model using only CCTA, both diagnostic models using CCTA with vessel-specific FAI or lesion-specific FAI demonstrated higher AUC (CCTA along, 0.607, 95% CI, 0.549–0.663; CCTA + vessel-specific FAI, 0.696, 95% CI, 0.640–0.748, *p* < 0.001; CCTA + lesion-specific FAI, 0.676, 95% CI, 0.619–0.729, *p* < 0.001). Additionally, both the vessel-specific FAI and lesion-specific FAI enabled the effective reclassification of CCTA diameter stenosis results as follows: CCTA + vessel-specific FAI (NRI, 49.6%, 95% CI, 28.1–70.4%, *p* < 0.001; relative IDI, 5.9%, 95% CI, 3.3–8.4%, *p* < 0.001), CCTA + lesion-specific FAI (NRI, 43.3%, 95% CI, 25.2–61.4%, *p* < 0.001; relative IDI, 5.4%, 95% CI, 2.9–7.8%, *p* < 0.001).

### 3.4. Relationship between Vessel-Specific FAI, Lesion-Specific FAI, and Coronary Stenosis

**Figure 5:**
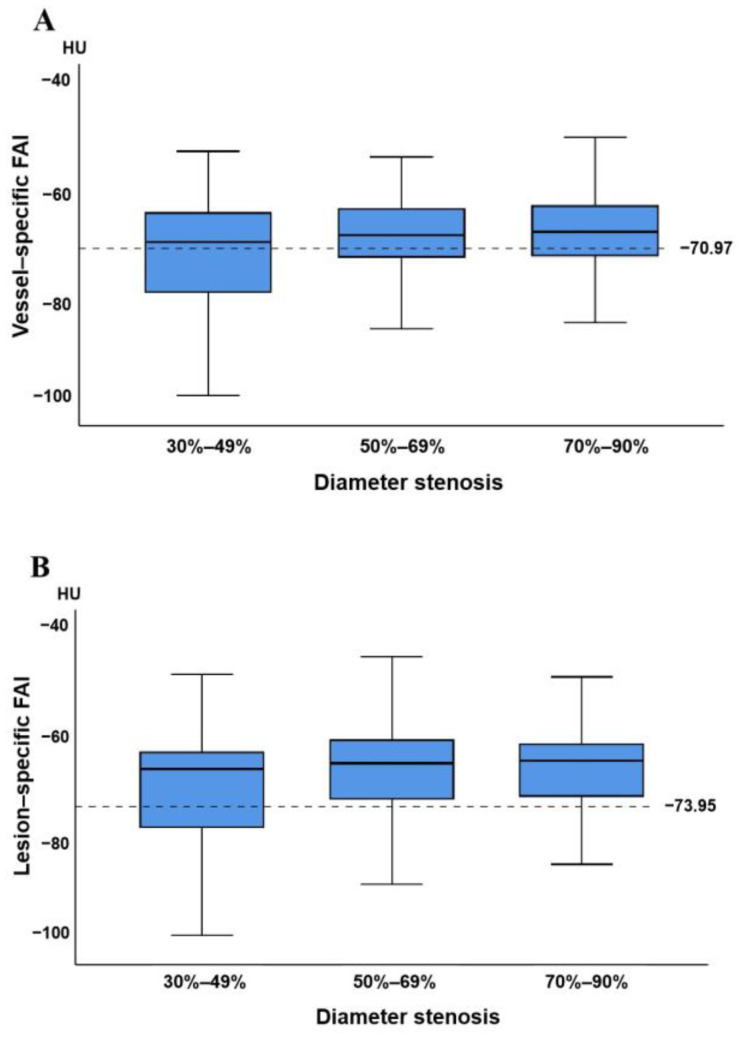
Relationship between vessel-specific FAI, lesion-specific FAI, and stenosis based on CCTA: (**A**,**B**) distribution of vessel-specific FAI (**A**) and lesion-specific FAI (**B**) scores in each group with 30–49%, 50–69%, and 70–90% diameter stenosis on CCTA. Medians, quartiles, and ranges of vessel-specific FAI and lesion-specific FAI scores are shown in the box plot. Cutoff values of vessel-specific FAI and lesion-specific FAI scores are displayed as dashed lines. FAI, fat attenuation index; CCTA, coronary computed tomography angiography.

The relationship between anatomical stenosis results determined by CT, vessel-specific FAI, and lesion-specific FAI is shown in Figure 5. Among the 233 vessels with obstructive stenosis (≥50%), vessel-specific FAI ≥ −70.97 HU accounted for 67.4% (157/233), while lesion-specific FAI ≤ −73.95 HU accounted for 79.8% (186/233). Among 119 severe stenosis (70–89%) lesions in CCTA, 75 (63.0%) demonstrated hemodynamic significance (invasive FFR ≤ 0.8), while moderate stenosis (50–69%) and mild stenosis (30–49%) accounted for 40.4% (46/114) and 19.7% (12/61), respectively.

### 3.5. Predictors of Vessel-Specific FAI and Lesion-Specific FAI

**Figure 6:**
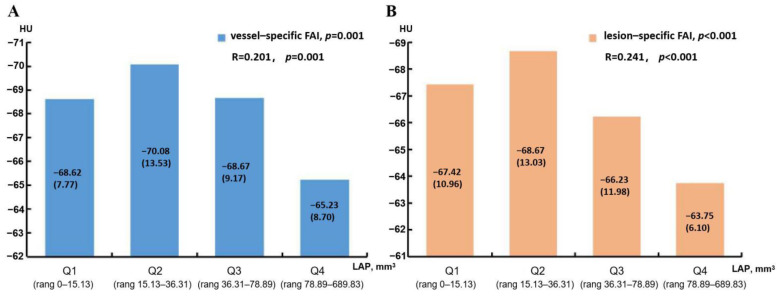
Distribution of vessel-specific FAI (**A**) and lesion-specific FAI (**B**) scores according to quartile of LAP volume (Q1–Q4). Values shown are medians (interquartile range). FAI, fat attenuation index; HU, Hounsfield unit; LAP, low-attenuation plaque.

As shown in Table 4, male patients tend to have higher vessel-specific FAI and lesion-specific FAI values (*p* = 0.024, *p* = 0.001, respectively). Smoking patients also tend to have higher lesion-specific FAI values (*p* = 0.032) rather than vessel-specific FAI values (*p* = 0.127). Patients with lesion-specific FAI ≥ −70.97 HU tend to have higher TPV, LAP volume, and plaque length values (*p* = 0.012, *p* = 0.002, and *p* = 0.010, respectively), while patients with lesion-specific FAI ≥ −73.95 HU just tend to have higher LAP volume values (*p* = 0.002). The results of the logistic regression are shown in Table 5. In the univariable analysis, men (OR, 1.832, 95% CI, 1.085–3.095, *p* = 0.024), TPV (OR, 1.002, 95% CI, 1.001–1.004, *p* = 0.009), LAP volume (OR, 1.009, 95% CI, 1.004–1.014, *p* = 0.001), and plaque length (OR, 1.018, 95% CI, 1.001–1.036, *p* = 0.034) were related to vessel-specific FAI scores. Additionally, men (OR, 2.708, 95% CI, 1.530–4.796, *p* = 0.001), smoker (OR, 1.821, 95% CI, 1.053–3.151, *p* = 0.032), and LAP volume (OR, 1.010, 95% CI, 1.003–1.016, *p* = 0.003) were related to lesion-specific FAI scores. In multivariable analysis, after adjustment, LAP volume was an independent risk factor for both vessel-specific FAI (OR, 1.008, 95% CI, 1.001–1.014, *p* = 0.016) and lesion-specific FAI scores (OR, 1.008, 95% CI, 1.003–1.014, *p* = 0.002). The relationships between the vessel-specific FAI, lesion-specific FAI, and LAP volume are shown in Figure 6, and both the differences in vessel-specific FAI and lesion-specific FAI between different LAP groups were statistically significant (*p* = 0.001, *p* < 0.001, respectively). Both the vessel-specific FAI (R = 0.201, *p* = 0.001) and lesion-specific FAI (R = 0.241, *p* < 0.001) scores were weakly correlated with LAP volume.

## 4. Discussion

The main findings of this study are as follows. No significant differences were noted between vessel-specific FAI and lesion-specific FAI scores, and there was a strong, almost collinear association between them. Secondly, the diagnostic AUCs and accuracy levels of vessel-specific FAI and lesion-specific FAI were not higher than for CCTA. However, the discrimination and reclassification ability for ischemia were significantly improved when vessel-specific FAI and lesion-specific FAI assessments, respectively, were added to CCTA. Lastly, the LAP volume was an independent risk factor for vessel-specific FAI and lesion-specific FAI values after adjusting for confounding factors.

Similar to previous studies [5,6], only 51.9% (121/233) of vessels with obstructive stenosis were hemodynamically significant in this study. Previous studies have also reported that lesion-specific ischemia diagnosed by FFR is associated with future adverse prognosis and that revascularization guided by FFR can improve event-free survival [19,20]. Consequently, this indicates that in addition to stenosis by CCTA, other non-invasive methods are needed to assist in improving the ability to discriminate ischemia.

Atherosclerosis is an inflammatory process [25] and inflammation is a key factor, not only for atherosclerotic development, but also for the progression of atherosclerotic plaques [26]. Perivascular FAI is a metric derived from CT, which reflects the presence of pericoronal inflammation [10]. The paracrine inflammatory signals from the inflamed vessel walls would prevent lipid accumulation by affecting biological processes such as adipocyte differentiation, proliferation, and lipolysis [9], thereby resulting in a shift from a lower to a higher water/lipid ratio, while the attenuation on CT images increases. Therefore, FAI would be higher when vascular inflammation occurs. Vascular inflammation is a chief contributor to endothelial dysfunction, leading to local “functional stenosis” [27,28]. FAI alone is a weak predictor of lesion-specific ischemia, because the diagnostic performance of the vessel-specific FAI or the lesion-specific FAI was not significantly higher than that of CCTA in this study. Compared to CCTA, the vessel-specific FAI or lesion-specific FAI aid in the identification of ischemia, resulting in relatively high-sensitivity and low specificity, rather than higher diagnostic AUC or accuracy. However, the discrimination and reclassification ability of hemodynamic significance stenosis were significantly improved when vessel-specific FAI and lesion-specific FAI assessments were added to CCTA, respectively, as reported previously [12,13,14,15]. Nevertheless, there were still some different results from previous studies, which may have been due to the different populations and software programs employed [29].

The measurement methods used for FAI in previous studies [12,13,14,15,29] were not consistent, which adds confusion in assessing the impacts of measurement methods on the diagnostic performance of the FAI. This study observed for the first time that there were no significant differences between vessel-specific FAI and lesion-specific FAI scores, and that there was a strong, almost collinear association between them. No significant difference in diagnostic performance was noted between the two. Therefore, the results of this study suggest that both FAI measurement methods could be applied to clinical practice. The vessel-specific FAI seems to be a more convenient and appropriate method than lesion-specific FAI because the former was measured automatically using software and it could reduce human error, meaning it had higher reliability. However, a large sample study is still needed for verification.

Patients with vessel-specific FAI scores ≥ −70.97 HU and lesion-specific FAI scores ≥ −73.95 HU tend to have higher LAP volumes. After adjustment, the LAP volume was shown to be an independent risk factor for vessel-specific FAI and lesion-specific FAI scores, and we also found that the LAP volume was positively correlated with both of these methods. Similar to our findings, Goeller et al. [30] demonstrated an increase in the burden of LAP marked by a significant increase in PVAT attenuation (*p* = 0.04), and there was an association between LAP burden and increased PVAT attenuation (R = 0.24, *p* = 0.01). LAP was the alternative to the presence of the necrotic core. The necrotic core causes inflammation and oxidative stress by improving the levels of vasoconstrictors and by reducing the production and bioavailability of vasodilators [28,31]. Moreover, vascular inflammation is associated with the FAI. Thus, the presence of necrotic cores is associated with the FAI, and this study supports these findings. In addition, CS is considered to be a specific marker of atherosclerotic burden, which might have a common inflammatory background with PVAT. A recent study speculated that there might be a potential correlation between PVAT and CS [32]. However, the results of this study found that there was no relationship between the FAI and CS, which may be related to the patient population and drug treatment received in this study.

This study still had some limitations. First, this study was a retrospective post hoc analysis of existing data. Thus, there may be potential selection bias in this study. Second, other CT-based techniques of functional assessment such as fractional flow reserve derived from computed tomography (FFR_CT_) have been widely introduced and used. Intraindividual comparisons need to be performed in the future to determine the best single or combination approach. Finally, this study lacks clinical outcome data, and further clinical outcome studies are still needed to analyze the effectiveness of these methods.

## 5. Conclusions

The FAI is an additional tool used to identify patients with relevant stenosis, and the combined use of a vessel-specific FAI or lesion-specific FAI and CCTA could improve the diagnostic performance of ischemia compared with CCTA alone. Thus, the need for further invasive treatment can be better assessed in patients. The LAP volume is the independent risk factor used for both tools.

## Figures and Tables

**Table 1:** Baseline characteristics of patients.

Characteristic	Value
Number of patients, *n*	261
Number of lesion vessels, *n*	294
Age, year	56.3 ± 8.7
Male, *n* (%)	188 (72.03)
Body mass index, kg/m^2^	25.9 ± 3.1
Risk factors	
Hypertension, *n* (%)	153 (58.6)
Diabete, *n* (%)	83 (31.8)
Dyslipidemia, *n* (%)	222 (85.1)
Smoker, *n* (%)	140 (53.6)
Family history of CAD, *n* (%)	47 (18.0)
Distribution of lesion	
LAD, *n* (%)	210 (71.4)
LCX, *n* (%)	35 (11.9)
RCA, *n* (%)	49 (16.7)
Calcification score	63.00 (3.18–208.45)
<400, *n* (%)	136 (52.1)
≥400, *n* (%)	125 (47.9)
CCTA stenosis	
Stenosis between 30–49%, *n* (%)	64 (21.77)
Stenosis between 50–69%, *n* (%)	114 (38.78)
Stenosis between 70–90%, *n* (%)	119 (40.48)
Invasive FFR	0.80 ± 0.11
Vessels with FFR ≤ 0.80, *n* (%)	133 (45.2)
RCA with FFR ≤ 0.80, *n* (%)	12 (4.1)
LAD with FFR ≤ 0.80, *n* (%)	46 (15.6)
LCX with FFR ≤ 0.80, *n* (%)	75 (25.5)

Note: Continuous variables are expressed as means ± standard deviation values or median (interquartile range) values, and categorical variables are expressed as numbers (percentages) of patients or lesions. CAD, coronary artery disease; LAD, left anterior descending coronary artery; LCX, left circumflex coronary artery; RCA, right coronary artery; CCTA, coronary computed tomography angiography; FFR, fractional flow reserve.

**Table 2:** Per-vessel diagnostic accuracy levels of CCTA, vessel-specific FAI, and lesion-specific FAI.

	True Positive ^a^	True Negative ^a^	False Positive ^a^	False Negative ^a^	% Accuracy	% Sensitivity	% Specificity	% PPV	% NPV	AUC
CCTA	121	49	112	12	57.82 (54.71–60.83)	90.98 (84.77–95.25)	30.44 (23.44–38.17)	51.93 (49.05–54.80)	80.33 (69.40–88.03)	0.607 (0.549–0.663)
Vessel-specific FAI ≥ −70.97 HU	104	75	86	29	60.88 (0.578–0.639)	78.20 (70.21–84.89)	46.58 (38.70–54.60)	54.74 (50.50–58.90)	72.12 (64.30–78.79)	0.677 (0.620–0.730)
Lesion-specific FAI ≥ −73.95 HU	118	53	108	15	58.16 (55.12–61.22)	88.72 (82.08–93.55)	32.92 (25.73–40.75)	52.21 (49.11–55.29)	77.94 (67.64–85.66)	0.665 (0.608–0.719)

Note: Except otherwise indicated, data are percentages with 95% confidence intervals. ^a^ Data are raw data. CCTA, coronary computed tomography angiography; FAI, fat attenuation index; PPV, positive predictive value; NPV, negative predictive value; AUC, area under curve.

**Table 3:** Comparison of different models for the identification of ischemia (FFR ≤ 0.80).

	AUC (95% CI)	Difference with CCTA (95% CI)	*p*	NRI (95% CI)	*p*	IDI (95% CI)	*p*
CCTA	0.607 (0.549–0.663)	-	-	-	-	-	-
CCTA + Vessel-specific FAI	0.696 (0.640–0.748)	0.089 (0.050–0.127)	<0.001	49.6% (28.1–70.4%)	<0.001	5.9% (3.3–8.4%)	<0.001
CCTA + Lesion-specific FAI	0.676 (0.619–0.729)	0.069 (0.036–0.102)	<0.001	43.3% (25.2–61.4%)	<0.001	5.4% (2.9–7.8%)	<0.001

Note: Data are calculated with 95% confidence intervals. FFR, fractional flow reserve; AUC, area under the curve; CI, confidence interval; NRI, net reclassification index; IDI, integrated discrimination improvement; CCTA, coronary computed tomography angiography; FAI, fat attenuation index.

**Table 4:** Patient and plaque characteristics according to vessel-specific FAI and lesion-specific FAI scores.

	Vessel-Specific FAI			Lesion-Specific FAI		
	≥−70.97 HU (*n* = 190)	<−70.97 HU (*n* = 104)	*p*	≥−73.95 HU (*n* = 226)	<−73.95 HU (*n* = 68)	*p*
Age, year	56.4 ± 8.8	56.2 ± 8.6	0.822	55.9 ± 8.8	57.9 ± 8.3	0.097
Male, *n* (%) ^a^	146 (76.84)	67 (64.42)	0.023	178 (78.76)	38 (55.88)	<0.001
Body mass index, kg/m^2^	25.86 ± 3.15	25.90 ± 3.02	0.911	25.86 ± 3.15	25.90 ± 2.96	0.920
hsCRP, mg/L	1.10 (1.57)	1.08 (1.74)	0.980	1.04 (1.57)	1.10 (1.75)	0.871
Hypertension, *n* (%) ^a^	113 (59.47)	60 (64.42)	0.767	133 (58.85)	40 (58.82)	0.997
Diabete, *n* (%) ^a^	56 (29.47)	34 (32.69)	0.567	66 (29.20)	24 (35.29)	0.339
Dyslipidemia, *n* (%) ^a^	160 (84.21)	89 (85.58)	0.756	193 (85.40)	56 (82.35)	0.541
Smoker, *n* (%) ^a^	109 (57.37)	50 (48.08)	0.126	130 (57.52)	29 (42.65)	0.031
Family history of CAD, *n* (%) ^a^	36 (18.95)	17 (16.35)	0.579	41 (18.14)	12 (17.65)	0.926
TPV, mm^3^	282.21 (215.82)	231.95 (218.76)	0.012	267.21 (216.64)	252.89 (234.30)	0.182
CP volume, mm^3^	17.38 (53.67)	15.89 (37.15)	0.963	15.53 (49.13)	18.41 (45.85)	0.308
IAP volume, mm^3^	182.83 (135.15)	168.87 (157.20)	0.112	182.32 (132.45)	173.34 (168.22)	0.442
LAP volume, mm^3^	49.70 (89.30)	30.65 (35.59)	0.002	44.63 (77.36)	28.60 (36.02)	0.002
CS	57.00 (212.35)	64.50 (154.15)	0.782	57.00 (188.58)	82.00 (314.40)	0.125
Plaque length, mm	24.28 (18.98)	18.71 (18.52)	0.010	23.59 (18.16)	18.29 (23.88)	0.054
PR, *n* (%) ^a^	57 (30.00)	30 (28.85)	0.836	70 (30.97)	17 (25.00)	0.344

Note: Unless otherwise indicated, data are means ± SD or medians (interquartile range). ^a^ Data are numbers (percentage). FAI, fat attenuation index; HU, Hounsfield unit; hsCRP, high-sensitivity C-reactive protein; CAD, coronary artery disease; TPV, total plaque volume; CP, calcified plaque; IAP, intermediate-attenuation plaque; LAP, low-attenuation plaque; CS, calcification score; PR, positive remodeling.

**Table 5:** Univariable and multivariable analysis for vessel-specific FAI and lesion-specific FAI.

**Univariable Analysis**				
	**Vessel-Specific FAI**		**Lesion-Specific FAI**	
	**OR (95% CI)**	***p***	**OR (95% CI)**	***p***
Age	1.003 (0.976–1.031)	0.821	0.973 (0.943–1.005)	0.097
Sex	1.832 (1.085–3.095)	0.024	2.708 (1.530–4.796)	0.001
Body mass index	0.996 (0.922–1.076)	0.911	0.995 (0.912–1.087)	0.919
hsCRP	1.025 (0.937–1.122)	0.586	1.067 (0.951–1.198)	0.267
Hypertension	1.076 (0.663–1.748)	0.767	1.001 (0.577–1.737)	0.997
Diabete	0.860 (0.514–1.440)	0.567	0.756 (0.426–1.343)	0.340
Dyslipidemia	0.899 (0.459–1.760)	0.756	1.253 (0.607–2.587)	0.541
Smoker	1.453 (0.899–2.349)	0.127	1.821 (1.053–3.151)	0.032
Family history of CAD	1.196 (0.635–2.255)	0.579	1.034 (0.509–2.102)	0.926
TPV	1.002 (1.001–1.004)	0.009	1.001 (1.000–1.003)	0.111
CP volume	1.002 (0.997–1.006)	0.405	0.999 (0.995–1.004)	0.789
IAP volume	1.002 (1.000–1.004)	0.063	1.001 (0.999–1.004)	0.292
LAP volume	1.009 (1.004–1.014)	0.001	1.010 (1.003–1.016)	0.003
CS	1.000 (1.000–1.001)	0.548	0.999 (0.999–1.000)	0.137
Plaque length	1.018 (1.001–1.036)	0.034	1.011 (0.992–1.029)	0.258
PR	1.057 (0.625–1.788)	0.836	1.346 (0.726–2.495)	0.345
**Multivariable Analysis**				
	**Vessel-Specific FAI**		**Lesion-Specific FAI**	
	**OR (95% CI)**	***p***	**OR (95% CI)**	***p***
Sex	1.541 (0.897–2.650)	0.117	1.478 (0.780–2.800)	0.231
Smoker	-	-	1.104 (0.616–1.980)	0.739
TPV	1.000 (0.997–1.002)	0.818	-	-
LAP volume	1.008 (1.001–1.014)	0.016	1.008 (1.003–1.014)	0.002
Plaque length	1.010 (0.989–1.032)	0.338	-	-

OR, odds ratio; CI, confidence interval; FAI, fat attenuation index; hsCRP, high-sensitivity C-reactive protein; CAD, coronary artery disease; TPV, total plaque volume; CP, calcified plaque; IAP, intermediate-attenuation plaque; LAP, low-attenuation plaque; CS, calcification score; PR, positive remodeling.

## Data Availability

Not applicable.
